# Immune Checkpoint Inhibitor-Induced Primary Adrenal Insufficiency: A Case Report

**DOI:** 10.5811/cpcem.20448

**Published:** 2024-08-26

**Authors:** Rahul Gupta, Cary Lubkin

**Affiliations:** Cooper University Hospital, Department of Emergency Medicine, Camden, New Jersey

**Keywords:** immunotherapy, shock, adrenal insufficiency, immune checkpoint inhibitor, case report

## Abstract

**Introduction:**

One of the less common and more life-threatening etiologies of adrenal insufficiency is immune checkpoint inhibitor (ICI)-induced primary adrenal insufficiency (PAI). Patients typically present with fatigue, malaise, and nausea and are treated empirically with hydrocortisone.

**Case Report:**

We present the case of a 59-year-old female who presented with hypotension, which initially was thought to be due to hypovolemia or medication-related, but was ultimately found to have PAI.

**Conclusion:**

This case highlights the importance of early detection of ICI-induced primary adrenal insufficiency, given its associated morbidity and mortality and its incidence in patients with a history of immunotherapy.

## INTRODUCTION

Adrenal insufficiency is a well-known cause of shock in patients, with various sub-etiologies. One of the less common etiologies includes immune checkpoint inhibitor (ICI)-induced primary adrenal insufficiency (PAI).^1^–^4^ Patients who are on ICIs are susceptible to many endocrine adverse events, many of which are thought to be secondary to autoimmune effects on various organs.^3^ Based on current literature, one of the rarer ICI-induced endocrinopathies is thought to be PAI.^2^ It is important to recognize and treat ICI-induced PAI early given the associated morbidity and mortality.^2^–^3^,^5^ Patients with ICI-induced PAI typically present with malaise, fatigue, and nausea.^1^–^2^,^6^

Evaluation of adrenocorticotropic hormone (ACTH) and cortisol levels is important when PAI is clinically suspected, although this should not delay empiric treatment with hydrocortisone in acutely ill patients.^1^,^5^–^6^ All ICI therapies have had some association with PAI. Immune checkpoint inhibitors are a relatively new form of immunotherapy and include the following: cytotoxic T-lymphocyte-associated protein inhibitor (eg, ipilimumab), programmed cell death protein 1 inhibitors (eg, nivolumab and pembrolizumab), and programmed cell death ligand 1 inhibitors (eg, atezolizumab, avelumab, and durvalumab).^3^ Given the rising use of ICI therapies, it is important that emergency physicians be aware of the incidence of ICI-induced PAI when evaluating a patient with undifferentiated shock with a history of treatment with these medications.

We describe a case spanning two emergency department (ED) presentations, coincidentally seen by the same resident and attending physician each time, and ultimately deemed to be ICI-induced PAI.

## CASE REPORT

A 59-year-old female presented to a tertiary ED with lightheadedness for one day. Per emergency medical services, the patient was found to be hypotensive by the home health aide, with a blood pressure of 60s/30s millimeters of mercury (mm Hg), but otherwise no mental status changes or other acute complaints. Of note, the patient had been discharged two weeks prior from an outside hospital after a two-week stay requiring ventilator support due to concern for a since-resolved cardiogenic shock state (ejection fraction 15–20%), thought to be secondary to stress-induced cardiomyopathy or secondary to use of ICIs, ipilimumab and nivolumab, which she had last received approximately two months before. She had a past medical history of hypertension, atrial fibrillation, hyperlipidemia, and mesothelioma for which she had recently been on ICIs, as discussed above. The patient reported no significant family or social history. Additionally, she was prescribed apixaban due to her atrial fibrillation. The patient was also on metoprolol succinate XL 50 milligrams (mg) daily and atorvastatin 40 mg daily.

On arrival, her blood pressure was 89/54 mm Hg, with a repeat blood pressure of 63/38 mm Hg. Other initial vitals were as follows: heart rate 55 beats per minute; temperature 36.6° Celsius (C); respiratory rate 18 breaths per minute; and oxygen saturation 96% on room air. The patient’s physical examination was notable for a chest wall port with no evidence of infection, but otherwise was benign. Electrocardiogram demonstrated sinus bradycardia to 55 with no changes from prior baseline. Point-of-care cardiac ultrasound demonstrated a mildly depressed ejection fraction, normal right ventricle/left ventricle ratio, and no pericardial effusion. No B-lines were visualized on point-of-care lung ultrasound.

Laoratory results were notable for lactic acid of 1.1 millimoles per liter (mmol/L) (reference range 0.5–2.2 mmol/L), hemoglobin of 11.2 grams per deciliter (g/dL) (14–18 g/dL), white blood cell count of 7.87x10^3^ per microliter (μL) (5.50–11x10^3^/μL), and 0- and 1-hour high sensitivity troponin trend was 59 to 46 nanograms (ng)/L (0–19 ng/L). Given the patient’s history of steroid use in the past (although it was unclear when it had last been taken) a random cortisol level was drawn and found to be 0.3 micrograms (μg)/dL (5.0–23.0 μg/dL). Basic metabolic panel had no electrolyte derangements. Her chest radiograph was unremarkable. The patient was started on broad spectrum antibiotics empirically given her hypotension with a potentially immunocompromised state.

The patient was given 500 milliliters of intravenous (IV) lactated Ringer’s with subsequent improvement in blood pressure to 119/58 mm Hg. She was subsequently admitted and was evaluated by the hospitalist and cardiology services, who believed hypotension was secondary to medications or hypovolemia. The patient was discharged the following day. Transthoracic echocardiogram (TTE) demonstrated an ejection fraction of 45–49% with mildly dilated left ventricle and left atrium. No changes were made to the patient’s medications.

The patient re-presented to the same ED seven days after her first presentation with hypotension and lightheadedness. On this visit, the patient reported chills and diarrhea but no other associated symptoms. Emergency medical services reported a blood pressure of 50/20 mm Hg en route. On arrival, she had a blood pressure 60/45 mm Hg, heart rate 87 beats per minute, temperature 36.7°C, respiratory rate 23 breaths per minute, and oxygen saturation 91% on room air. Point-of-care ultrasound showed no change from prior TTE. Computed tomography of the chest and abdomen/pelvis were significant for trace left pleural effusion, small right pleural effusion with baseline pleural thickening, and enlarged subcarinal lymph nodes. After no improvement with two liters of IV lactated Ringer’s, the patient was started on norepinephrine with a mean arterial pressure goal of 65 mm Hg, necessitating an infusion at a rate of 15 μg/kilogram (kg) per minute (min). As at the previous visit, she was empirically started on broad spectrum antibiotics. There were no significant laboratory findings including the basic metabolic panel with no electrolyte derangements. Baseline cortisol drawn at the time of her admission was 1.7 μg/dL (reference range 5.0–23.0 μg/dL).

**CPC-EM Capsule**

What do we already know about this clinical entity?

*Immune checkpoint inhibitors (ICI) can cause primary adrenal insufficiency, a rare but life-threatening condition typically presenting with fatigue, malaise, and nausea.*

What makes this presentation of disease reportable?

*This presentation of primary adrenal insufficiency is due to ICIs, a treatment that is becoming more prevalent.*

What is the major learning point?

*It is vital to consider primary adrenal insufficiency as a cause of shock in patients who are currently being treated with ICIs.*

How might this improve emergency medicine practice?

*Being aware of this life-threatening condition will allow emergency physicians to recognize and treat the condition earlier in its course.*

After intensive care unit admission, evening cortisol was found to be 0.3 μg/dL. Endocrinology was consulted, and as per their recommendations a cosyntropin stimulation test was performed without an appropriate response: 30-min cortisol = 6.4 μg/dL (>18 μg/dL) and 60-min cortisol = 8.6 μg/dL (>18 μg/dL). Adrenocorticotropic hormone (ACTH) noted to be elevated. Based on these results, endocrinology was concerned for PAI due to ICI use and stress dose hydrocortisone 50 mg every eight hours was initiated with improvement in baseline blood pressure and decreasing pressor requirements. This dose was tapered, and the patient was subsequently discharged on hydrocortisone 15 mg every morning and 5 mg nightly. She did later have a repeat admission for hypotension secondary to running out of hydrocortisone at home, but her blood pressure quickly improved with the administration of hydrocortisone and she was discharged the day after admission with no complications.

## DISCUSSION

This case demonstrates a case of ICI-induced PAI, a rare complication of ICI therapy. The pathophysiology is poorly understood, but it is thought to be secondary to T-cell mediated destruction of the adrenal cortex.^1^–^2^ Laboratory testing will typically reveal decreased random or morning cortisol levels, inappropriate cosyntropin stimulation test response, and elevated ACTH levels.^1^,^5^–^6^ If ACTH is noted to be decreased, secondary adrenal insufficiency should be considered, which is postulated to be due to ICI-induced hypophysitis, inflammation of the pituitary gland.^6^ Electrolyte derangements may also occur if mineralocorticoid-producing cells are also affected, resulting in hyperkalemia and hyponatremia.^1^,^5^ Patients typically present with malaise, fatigue, and nausea, with associated hypotension, much like the patient in this case.^1^–^2^,^6^ As these patients are often immunocompromised and present with undifferentiated shock, it is important to thoroughly rule out other causes of shock, including septic shock and hypovolemic shock.

Treatment typically consists of corticosteroid therapy immediately with consideration for mineralocorticoid therapy if there is evidence of hyperkalemia and hyponatremia, which demonstrates involvement of mineralocorticoid-producing cells. Given the life-threatening nature of ICI-induced PAI, if there is a heightened clinical suspicion for this condition, it is vital to treat empirically in the ED with corticosteroids while laboratory tests are pending for confirmation of diagnosis. Unfortunately, though, it is common for the diagnosis to be delayed given the initially insidious nature and non-specific symptomatology.^2^ Per the current literature, the recommended treatment is hydrocortisone as an empiric treatment in critically ill patients.^1^–^2^,^6^ Typically, an initial dose of 100 mg hydrocortisone IV should be administered followed by 50 mg IV every six hours. If available, an endocrinology consult should be obtained for further guidance.^5^

Ultimately, ICI-induced PAI is a very rare complication of ICI therapy. In their literature review in 2018, Barroso-Sousa et al reported 43 total cases of any-grade PAI among 5,381 patients given ICI monotherapy, representing an incidence of 0.7%.^3^ They also found the rate of PAI in all patients treated with combination ICI therapy, as in our patient, to be 4.2%.^3^ Other studies report 451 instances found in the literature of ICI-induced PAI, but report that only 45 of them were definite, laboratory-confirmed cases while the others were assumed cases.^7^–^8^ Interestingly, more than 90% of the cases of ICI-induced adrenal insufficiency are classified as secondary adrenal insufficiency.^9^

Ultimately, the incidence of ICI-induced PAI is rare but varies widely, depending on the type and dose of ICI. Further complicating the diagnosis is the variability of the timeline that patients may present with ICI-induced PAI, with a median onset of about 10 weeks. There are also reported cases ranging from 2–4 months, and many cases are noted to be found long after the end of treatment, as in this patient.^10^–^13^

## CONCLUSION

Emergency physicians often encounter patients in a shock state and are well-versed in differentiation and treatment of the most common causes of shock. Unfortunately, there are rare occasions of refractory shock where other diagnoses and etiologies should be considered. Given the rising use of immune checkpoint inhibitor therapy, ICI-induced primary adrenal insufficiency is a diagnosis that should be considered when there is a high clinical suspicion and treated expeditiously given the life-threatening nature of the disease.
